# Glucose Regulates Rat Beta Cell Number through Age-Dependent Effects on Beta Cell Survival and Proliferation

**DOI:** 10.1371/journal.pone.0085174

**Published:** 2014-01-09

**Authors:** Zerihun Assefa, Astrid Lavens, Christophe Steyaert, Geert Stangé, Geert A. Martens, Zhidong Ling, Karine Hellemans, Daniel Pipeleers

**Affiliations:** Diabetes Pathology and Therapy Unit, Diabetes Research Center and Center for Beta Cell Therapy, Brussels Free University-VUB, Brussels, Belgium; Sapienza University of Rome, Italy

## Abstract

**Background:**

Glucose effects on beta cell survival and DNA-synthesis suggest a role as regulator of beta cell mass but data on beta cell numbers are lacking. We examined outcome of these influences on the number of beta cells isolated at different growth stages in their population.

**Methods:**

Beta cells from neonatal, young-adult and old rats were cultured serum-free for 15 days. Their number was counted by automated whole-well imaging distinguishing influences on cell survival and on proliferative activity.

**Results:**

Elevated glucose (10–20 versus 5 mmol/l) increased the number of living beta cells from 8-week rats to 30%, following a time- and concentration-dependent recruitment of quiescent cells into DNA-synthesis; a glucokinase-activator lowered the threshold but did not raise total numbers of glucose-recruitable cells. No glucose-induced increase occurred in beta cells from 40-week rats. Neonatal beta cells doubled in number at 5 mmol/l involving a larger activated fraction that did not increase at higher concentrations; however, their higher susceptibility to glucose toxicity at 20 mmol/l resulted in 20% lower living cell numbers than at start. None of the age groups exhibited a repetitively proliferating subpopulation.

**Conclusions:**

Chronically elevated glucose levels increased the number of beta cells from young-adult but not from old rats; they interfered with expansion of neonatal beta cells and reduced their number. These effects are attributed to age-dependent differences in basal and glucose-induced proliferative activity and in cellular susceptibility to glucose toxicity. They also reflect age-dependent variations in the functional heterogeneity of the rat beta cell population.

## Introduction

Glucose is since long considered as regulator of the beta cell mass [1], [2], [3], [4]. The nutrient can influence survival and replication of beta cells, two mechanisms that can independently cause changes in beta cell number. However, its effects can be negative or positive depending on the experimental conditions, sometimes leading to conflicting data.

Several studies have reported glucotoxicity at prolonged supraphysiologic concentrations [5]; since they mostly used beta cell functions as parameter it was not clear to which extent toxicity reflected cell dysfunction or cell loss. In cultures of rat beta cells we have previously observed increased percentages of dead cells at and beyond 20 mmol/l glucose [6]; the highest survival rate was measured at 10 mmol/l glucose, the concentration that also maintains glucose-responsive beta cell functions [7]. At lower concentrations, beta cells lost their differentiated gene expression and progressively died in apoptosis, reflecting a role of glucose as survival factor that activates synthesis of anti-apoptotic proteins [5], [8].

In terms of beta cell replication, glucose was shown to increase proliferative activity in beta cells during short incubations [9], [10], but changes in beta cell number were not reported. This was also the case following glucose infusion in rodents [11], [12]. It is still unclear whether the in situ beta cell mass increases under sustained hyperglycemia or decreases as result of glucotoxicity. In transgenic mice with conditional but variable ablation of their pancreatic beta cells, all animals exhibited higher percentages of proliferating beta cells, also those with near normal glycemia [13]; the proliferation activation was attributed to a sustained intracellular “work load” involving chronic stimulation of glucokinase and glycolysis [13], [14]. Since the latter mechanism also induces insulin release, it is to be examined whether secretory-responsive cells are also proliferation-responsive. Data might further illustrate the functional heterogeneity within the beta cell population [15] and they can also indicate whether glucose acts as mitogen [16] or as permissive factor for other beta cell proliferation inducers [17], [18].

In vivo models are certainly adequate to identify regulators of beta cell mass in physio(patho)logic conditions. Their study design and interpretation can benefit from in vitro data demonstrating effects of specific agents on the number of cells. To this end, we developed a method for following the number of living beta cells during two weeks of culture without serum, and hence its survival and mitogenic factors. During this period, influences on beta cell survival were analyzed by vital staining, those on beta cell proliferation by thymidine-analog incorporation and cell number counts. The effect of glucose was examined in young-adult cells at concentrations that were previously found to recruit beta cells into metabolic and biosynthetic activity [19]. Since beta cells with higher glucose sensitivity exhibited a higher glucokinase activity [20], we investigated whether a glucokinase activator helps recruit beta cells into proliferation. Our in vitro study can thus provide direct support for this mechanism and localize the responsive cells within the functional heterogeneity of the beta cell population [15].

## Materials and Methods

### Ethics Statement

All experiments were approved by the Ethical Committee of the Vrije Universiteit Brussel and conducted according the European Community Council Directive (86/609/EEC).

### Materials

Culture medium and supplements were purchased from Invitrogen (Life Technologies Ltd, Paisley, UK), glucokinase activator, RO-28-1675, from Axon Medchem (Groningen, The Netherlands). Anti-insulin antibody was prepared in our laboratory, anti-bromo-deoxyuridine (BrdU) antibody purchased from DakoCytomation (Glostrup, Denmark) and Hoechst 33342 and propidium iodide from Sigma. The 804G cell line and the method for preparation of its matrix have been described before [21]. The cells were kindly provided by Dr T. Otonkoski, University of Helsinki, Finland. Isolated beta cells were seeded in 804G matrix-coated 384-well black plates (Greiner Bio-One, Frickenhausen, Germany).

### Purification and culture of rat beta cells

Beta cells were FACS-purified from islet-cell enriched suspensions [22] prepared from neonatal (2–3 days-old) and adult (8 or 40-week-old, male) Wistar rats (Janvier Bioservices, France); their purity was, respectively 70–75% and over 90%. Sorted cells were counted in a hemocytometer, seeded (day 0) and cultured for up to 15 days in serum-free Ham's-F10 medium at 5, 10 or 20 mmol/l glucose, supplemented with 2 mmol/l L-glutamine, 50 µM 3-isobutyl-1-methylxanthine, 0.5% Albumax-I, penicillin (100 U/ml) and streptomycin (0.1 mg/ml). The 10 mmol/l glucose condition is known to maintain beta cell survival over 9-days [6], [8]. Medium and cells were retrieved after selected culture periods and assayed for insulin content; data were expressed as a function of the number of living beta cells that was determined in parallel wells.

### Expression of selected mRNAs by quantitative real-time PCR

The mRNA expression level of selected genes was determined by real-time PCR using hydrolysis probe obtained from Applied Biosystems (TaqMan MGB, assays' IDs available on request) and analyzed after normalization with 5 reference genes (psmc5, ppia, rplp2, hprt1 and ubc) as described before [23], [24].

### Measurement of number, survival and proliferation activity in cultured beta cells

On day 1, 9 and 15 of culture, total numbers of living beta cells per well were determined by semi-automatic cell count, after Hoechst 33342 (Ho) and propidium iodide (PI) staining. Numbers of living beta cells were obtained by subtracting the PI-positive cell numbers from the Ho-positive cell numbers, and correcting for the percent insulin-negative cells, as determined following insulin-immunofluorescence staining. They were expressed as percent of the numbers on day 1. Each condition was also evaluated for increases in the percentage of proliferating beta cells (incorporating the thymidine-analogue 5-ethynyl-2′-deoxyuridine, EdU; 100 µmol/l). To detect beta cells that underwent two successive cycles, cells were sequentially exposed to two different thymidine-analogs, first EdU from day 3 to 6, and then BrdU (100 µmol/l) from day 7 to 10, with a 24-hour label-free period in between. EdU-positive (EdU^+^) nuclei were detected with the Click-iT Assay kit (Invitrogen), while BrdU-positive (BrdU^+^) nuclei were identified with anti-BrdU antibody.

### Data acquisition and analysis

Absolute number of cells was determined by whole-well imaging using the BD-PathwayBioimager855 (BD-Biosciences, [25]). IPLab and AttoVision software packages (BD-Biosciences) were used for image background subtraction, segmentation and quantification. The method was validated for 96- and 384-well plates in which living and dead cells, insulin-positive cells and nuclei with thymidine-analogues were counted.

### Statistics

Data are presented as means ± standard error of the mean (SEM) of n independent experiments. Each condition consisted of triplicate samples and was tested at least three times. Statistical differences between means were assessed with two-tailed unpaired Student's *t*-test or ANOVA with Tukey's test for multiple comparisons using GraphPad Prism (GraphPad Software, San Diego, USA). Differences were considered significant at *p*<0.05.

## Results

### Proliferation assay by imaging-based cell counts

**Figure 1:**
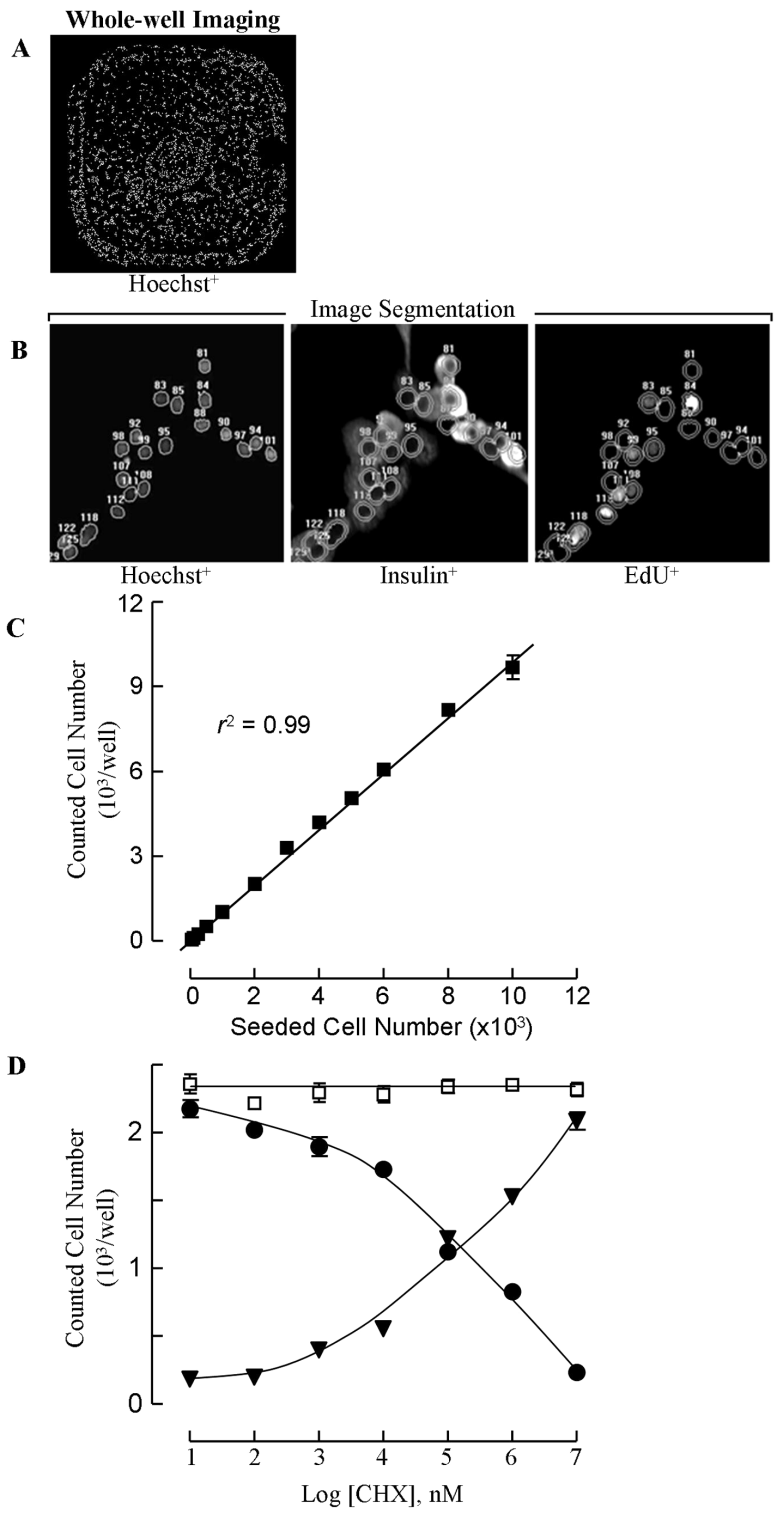
Image analysis for counting the number of living and dead beta cells per well and determining the percentages of EdU-positive cells. Purified rat beta cells were seeded in 96- or 384-well plates and stained by Hoechst 33342 (Ho) and propidium iodide. Whole-well images were taken at a resolution that allows identification and counting of all cells distinguishing PI-positive and PI-negative cells (A,C,D). IPLab and AttoVision software packages were used for image processing and quantification. Following image segmentation, every cell is numbered and the number of living (Ho-pos, PI-neg) and dead cells (Ho-pos-PI-pos) are determined. Induction of apoptosis by cycloheximide-CHX (8) causes within 48 h a dose-dependent increase in the number of dead cells (black triangles) and decrease in the number of living cells (black circles) without change in the total numbers (while squares) that are counted (D) (means ±SEM of three independent experiments). Image segmentation delineates nuclear and cytoplasmic boundaries of every cell through dynamic thresholding that detects changes in Hoechst fluorescence above background; the cytoplasm was designated by the area between the nuclear boundary and a three pixel-wide annular ring drawn around it (B). Following staining for insulin and EdU, the nuclear image is superimposed on the EdU and insulin immunofluorescence images and fluorescence intensity is averaged for pixels within the nuclear or cytoplasmic area so that the percentages of single or double-positive cells can be determined.

In order to validate imaging-based cell counts, a known number of beta cells, as determined in the hemocytometer, was seeded in 384-well plates and semi-automatically counted after one day of culture. Whole-well images were acquired and quantified by an operator-independent system as described in Fig. 1. A linear correlation was found between seeded and counted cell numbers (adjusted r^2^ = 0.99) over a large range (100 to 10,000 cells per well; Fig. 1C). Above 10,000 cells per well, precision decreased since cell aggregation limited segmentation of individual cells. Subsequent experiments were therefore conducted with 2000 cells seeded per well (18000 cells/cm^2^). The method distinguishes the numbers of living and of dead cells using propidium iodide as marker for dead cells. When cells were cultured for 2-days with cycloheximide, a known inducer of apoptosis in beta cells [8], an inverse correlation was found between the numbers of living and dead cells, while the total numbers remained constant (Fig. 1D). These results indicate that the assay can detect conditions of beta cell death and assess their influence on the number of living beta cells in culture.

### Glucose induces dose-dependent increase in number of adult rat beta cells during culture

Two weeks culture at 5 mmol/l glucose decreased the number of living beta cells by 21 percent (Table 1). This effect can be attributed to apoptosis which occurs during culture at this glucose concentration but not at 10 mmol/l [8]; this is supported by the higher percent dead cells counted at day 15. Culture at 10 and 20 mmol/l glucose increased beta cell numbers by, respectively, 16 and 31 percent (Table 1). This increase was time-dependent; after 9 days, it was not yet observed at 10 mmol/l, while in part present at 20 mmol/l glucose (21 percent increase). It not only reflects glucose-induced suppression of apoptosis but also induction of replication. There was no increase in the number of insulin-negative cells (>88% insulin-positive cells on day 15); an increase in total cell number is thus indicative for beta cell replication.

**Table 1:** Effect of glucose on the number of living beta cells.

Culture Condition	Number of Living Beta Cells (% of day 1)		
	Day 1	Day 9	Day 15
5 mmol/l glucose	1744±15	1503±100	1376±93**,≠
	(100±3)	(86±6)	(79±5)
10 mmol/l glucose	1734±15	1897±56	2027±56**
	(100±4)	(109±3)	(116±3)
20 mmol/l glucose	1752±15	2126±123**	2308±72***,#
	(100±3)	(121±7)	(131±4)

Adult rat (8-week-old) beta cells were cultured in serum-free medium at the indicated concentrations of glucose. The numbers of viable beta cells are expressed as absolute cell number per well and as percent of the numbers on day 1. Data represent means ± SEM; compared with day 1 values: *, p<0.05; **, p<0.01; ***, p<0.001; compared with 10 mmol/l glucose condition on day 15: ≠, p<0.001; #, p<0.01; n = 8.

### Glucose-induced recruitment of adult rat beta cells into DNA-synthetic activity reveals heterogeneity in cell responsiveness

**Figure 2:**
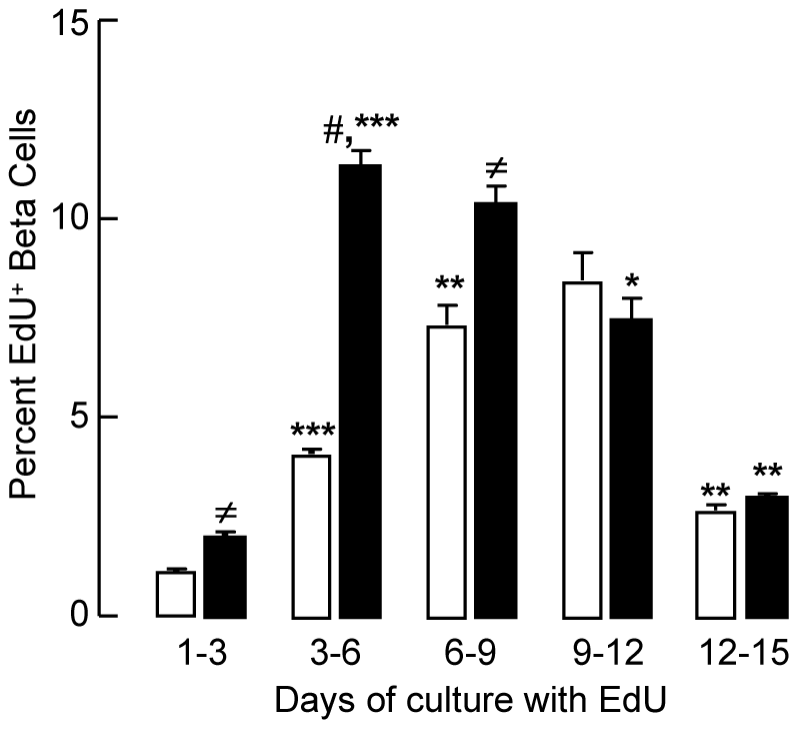
Effect of glucose on percent beta cells in DNA synthesis. Beta cells from 8-week old rats were cultured for the indicated periods at 10 mmol/l (white bars) or 20 mmol/l (black bars) glucose in presence of EdU. At the end of each period, the percent EdU^+^Insulin^+^ cells is determined. Data represent means ± SEM; ≠, p<0.05; #, p<0.001 10 mmol/l versus 20 mmol/l; *, p<0.05; **, p<0.01; ***, p<0.001 versus preceding period; n = 3.

To evaluate the kinetics of glucose-induced proliferation, beta cells seeded at 10 or 20 mmol/l were cultured with the thymidine-analogue EdU for selected time slots (48 h at start between day 1 and 3, and 72 h time slots thereafter, Fig. 2). At 10 mmol/l glucose, percent EdU^+^ beta cells was low on day 3 (1.1±0.1%), time-dependently increased up to 8.5±0.7% on day 12 and then decreased (to 2.7±0.1% on day 15). At 20 mmol/l, the percentage was only slightly higher on day 3 (2.0±0.2%) but three-fold on day 6 (11.3±0.4%) reaching its maximum and maintaining it during the subsequent three days; it then decreased to similar levels as at 10 mmol/l (Fig. 2). The sum of the percentages counted during the subsequent intervals (24% at 10 and 34% at 20 mmol/l glucose) could overestimate the total percent recruited cells as it might contain two EdU-positive daughter cells for cells that have been activated during the prior period. On the other hand, some daughter cells may have re-entered a new cycle, and should thus be distinguished from activated quiescent cells. The data on EdU incorporation are nevertheless in line with the observed increases in cell number (Table 1). Moreover, they provide evidence that glucose only recruited a fraction of beta cells into DNA-synthesis. This recruitment required several days of exposure to stimulating glucose concentrations with a more rapid effect at 20 mmol/l.

To examine whether newly formed beta cells were the progeny of a small pool of repetitively replicating cells or a result of time-dependent recruitment of new beta cells into the cell cycle, we performed sequential labeling with two thymidine-analogues [26]. It has been previously shown that prior incorporation of a thymidine-analog in beta cells does not diminish their probability for cell cycle re-entry [26], [27], which we confirmed in INS1832/13 cells (data not shown). Purified rat beta cells were first incubated with EdU from day 3 to 6, and then with BrdU from day 7 to 10. The percent EdU^+^ cells that were BrdU^+^ on day 10 represents the fraction of cells that re-entered the cell cycle during the second period. This fraction was only 0.5±0.1% of all cells at 10 mmol/l and 1.1±0.2% at 20 mmol/l glucose, far less than the percentages that had entered the cell cycle during the first three day period (respectively, 5.8±0.4% and 8.1±1.2%; Table 2). Cell cycle re-entry within 3 days is thus a rare event in the present conditions. On the other hand, the corresponding percentages of BrdU^+^ cells (8.8±0.7 and 17.7±2.3%) indicate that glucose continues to recruit beta cells into proliferation during this second period, mostly from the subpopulation that was not activated during the first period and more so at 20 mmol/l glucose. It is thus concluded that the time-dependent increase in the number of beta cells (Table 1) does not result from a small pool of repetitively replicating cells but mainly from recruitment of progressively more quiescent cells into the cell cycle.

**Table 2:** Effect of glucose on recruitment of beta cells into DNA-synthesis and on cell cycle re-entry of previously recruited cells.

Culture Condition	Day 6	Day 10		
	% EdU^+^	% EdU^+^BrdU^−^	% BrdU^+^EdU^−^	% EdU^+^BrdU^+^
Adult beta cells				
10 mmol/l glucose	5.8±0.4	8.0±0.4	8.3±0.7	0.5±0.1
20 mmol/l glucose	8.1±1.2	9.5±1.6	16.6±2.1**	1.1±0.2*
Neonatal beta cells				
10 mmol/l glucose	28.7±2.2***	27.1±3.7***	23.1±5.2**	11.3±1.6**

Adult (8-week-old; n = 5) and neonatal (2–3 days-old; n = 3) beta cells were cultured at the indicated glucose concentrations for 10 days with sequential labeling with EdU (day 3 to day 6) and BrdU (day 7 to day 10). The percentages of EdU^+^ and/or BrdU^+^ beta cells are shown as means ± SEM; compared with adult 10 mmol/l glucose: *, p<0.05; **, p<0.01; ***, p<0.001.

In view of the higher percent double-labeled cells at 20 mmol/l glucose, we examined whether this reflects a shortened cell cycle re-entry period as was suggested by observations in vivo [27]. A mathematical model [26] was used to estimate the probability that a replicated beta cell re-enters the cell cycle. To this end, the fraction of EdU^+^ beta cells at day 10 was multiplied by the corresponding fraction of BrdU^+^ cells. If beta cell replication occurs stochastically, this predicted value should equal the counted percent EdU^+^BrdU^+^ beta cells; it will be lower if the cell cycle re-entry period is shortened, as in a continuously replicating subpopulation. Our data suggest that the counted fractions of EdU^+^BrdU^+^ cells were significantly lower than the predicted values, both at 10 and 20 mmol/l glucose (Fig. S1), indicating a prolonged refractory period in both conditions. The higher fraction of double-labeled beta cells at 20 mmol/l glucose is thus attributable to a higher number of cells that has been recruited into the S-phase, as is also reflected by the higher percent of only BrdU^+^ cells in this condition (Table 2).

### Glucokinase activation recruits adult rat beta cells into DNA-synthesis but does not increase the pool of beta cells that is recruitable by glucose

**Figure 3:**
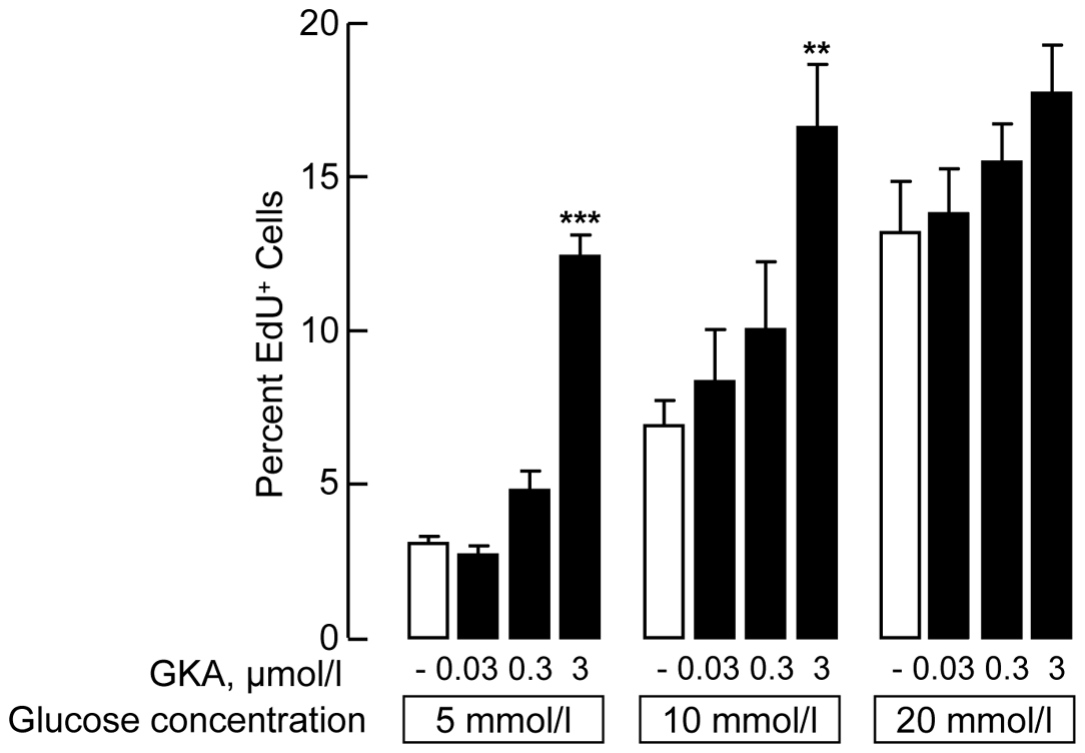
Effect of glucokinase activator (GKA) on recruitment of beta cells into DNA synthesis. Beta cells purified from 8-week old rats were cultured for six days at the indicated glucose concentrations in absence or presence of GKA. EdU was added during the last three days and the percent EdU^+^Insulin^+^ cells determined on Day 6. Data represent means ± SEM; **, p<0.01; ***, p<0.001 versus corresponding condition without GKA, n = 4.

Since intercellular differences in glucose sensitivity are associated with differences in glucokinase level and activity [20], [28], we examined whether a small-molecule glucokinase activator (GKA, RO-28-1675) increases the sensitivity of beta cells to a glucose-induced activation of DNA-synthesis. This compound was shown to increase the glucose affinity and V_max_ of glucokinase in cultured rat islets [29]. At both 5 and 10 mmol/l glucose, the presence of 3 µmol/l GKA increased the percent beta cells in DNA-synthesis to the levels measured at 20 mmol/l glucose; when added to the 20 mmol/l condition, no further increase was seen (Fig. 3). These data indicate that glucokinase activation reduces the threshold for glucose-induced recruitment of beta cells into DNA-synthesis but does not increase the percentage of beta cells that is maximally recruitable by glucose into this activity.

### Characteristics of adult beta cell population following glucose-induced recruitment into replication

We examined whether the condition for glucose-induced replication of adult beta cells had induced changes in characteristics of the population. This analysis was limited to measurement of cellular insulin content and release at end of culture, and simultaneous quantification of beta cell characteristic genes.

Cellular hormone content and release over 72 hours were measured on day 15 (after cell numbers had increased) and compared to data obtained before replication (day 3) whereby all values were expressed per 10^6^ cells that were counted on these days. The increases in beta cell number at 10 and 20 mmol/l glucose (day 15) were not associated with a reduced insulin releasing or storage capacity of the populations (Table 3). In fact, cellular insulin content after two weeks at 10 mmol/l glucose (30.6±3.6 ng insulin/10^3^ cells) was two-fold higher than at day 3, and approached the values in freshly purified rat beta cells (44.1±3.1 ng insulin/10^3^ cells). The reduction in cellular insulin content during the first 3-days of culture was thus restored during the subsequent 12 days. This correction did not occur at 20 mmol/l where a higher proportion of insulin was discharged (per hour, 10.3% of content versus 5.7% at 10 mmol/l glucose) and probably accounted for the lack of regranulation under this chronically elevated glucose concentration.

**Table 3:** Comparison of insulin release and cellular insulin content before and after glucose-induced replication.

Culture Condition	Cell Number (% of day 1)	Insulin Release (ng/72 h/10^3^ cells)	Insulin Content (ng/10^3^ cells)
Day 3			
10 mmol/l glucose	101±4	121±11	16±3
20 mmol/l glucose	103±5	136±10	14±2
Day 15			
10 mmol/l glucose	117±7*	122±13	31±4**
20 mmol/l glucose	143±15**	149±44	20±6

Insulin released into culture medium from day 0 to 3 and from day 12 to 15 and also in extracts cells collected on day 3 and day 15 was measured. Data was expressed as function of the number of cells counted on these days. Data represent means ± SEM; *, p<0.05; **, p<0.01 versus corresponding day 3 values; n = 4.

Expression of beta cell characteristic genes was analyzed after 15 days culture at 10 mmol/l glucose and showed comparable mRNA levels of Ins1, Ins2, Pdx1, Nkx6.1, PCSK1, Glut2 and Gck to those in freshly purified cells (Fig. S2).

### Effect of age on glucose-induced increases in beta cell number

We examined whether the effects of glucose observed in beta cells from young-adult rats (8-weeks) were also seen in beta cells from neonatal and old-adult rats (40-week; Table 4). The number of living beta cells from 40-week-old rats did not vary significantly over the two-week culture period; in contrast to the 8-week-old rats, there was no decrease at 5 mmol/l glucose and no increase at 10 and 20 mmol/l. On the other hand, the number of living neonatal beta cells doubled at 5 and 10 mmol/l glucose and decreased by 19 percent at 20 mmol/l (Table 4); the following observations can explain the marked changes in neonatal cells.

**Table 4:** Age-dependency of glucose effect on number of living rat beta cells.

	Number of Living Beta Cells on Day 15 (% of day 1)		
Age (weeks)	0	8	40
Culture 15 days			
5 mmol/l glucose	193±12*	74±5**	93±9
10 mmol/l glucose	198±12*,≠	118±5**	106±6
20 mmol/l glucose	81±14*,#	127±5***	110±11

Beta cells isolated from the different age groups were cultured at the indicated concentrations of glucose and the numbers of viable beta cells were determined after 15 days in culture and expressed as percent of the numbers on day 1. Data represent means ± SEM; *, p<0.05; **, p<0.01; ***, p<0.001 versus corresponding day 1 values; **≠**, p<0.05; **#**, p<0.01 versus 8-week-old at 10 mmol/l glucose; n = 4–8.

Real time PCR analysis of freshly isolated neonatal cells showed lower mRNA levels for Glut2, Ins1, Ins2 and MafA than in 8- and 40-week-old rats, which can be related to their immature state [30]; their glucokinase mRNA levels were however comparable (Fig. S3). When cultured at 10 mmol/l glucose, significantly larger proportions of neonatal cells were consecutively recruited into proliferative activity as shown by EdU-BrdU sequential labeling (Table 2): 28.7±2% EdU^+^ cells between day 3 and 6, and 23.1±5.2% BrdU^+^EdU^−^ cells between day 7 and 10, together 52% versus only 14% in beta cells from 8-week-old rats (Table 2). In addition, a markedly larger fraction of cells activated during the first phase re-entered the cell cycle during the second period (24% of EdU^+^ neonatal cells on day 10 were also BrdU^+^ versus only 5% in young-adult beta cells). When expressed as percentage of total beta cell number on day 10, 11% of neonatal beta cells had participated in at least two replicative cycles, 20-fold more than in young-adult beta cells (Table 2). However, when comparing the predicted and counted values for double-positive cells, no difference was found suggesting that neonatal beta cells that had been recruited into proliferation were not preferentially activated towards re-entry nor have they been primed for a prolonged refractory period (Fig. S1). The higher fraction of neonatal beta cells that re-enters the cell cycle is thus a consequence of the higher percent cells that enter proliferative activity. This was the case for 50% of the neonatal beta cells, which is in line with the doubling in cell number. Since a similar increase in beta cell number was seen at basal glucose concentration (5 mmol/l), there is no evidence that this replication is the result of a dose-dependent glucose-regulated recruitment of beta cells into proliferation, as was the case for the young-adult beta cell population. Neonatal beta cells were previously also found to exhibit a higher insulin biosynthetic and secretory activity at basal glucose levels [24].

The decrease in the number of living neonatal beta cells at 20 mmol/l was associated with a high percentage of dead cells at the end of culture, much more than in the conditions with young- and old-adult beta cells (Table S1). Measurement of a high percent dead cells at day 15 is a sign of a cytotoxic process during culture, and thus an indication that a number of cells may have disintegrated before this analysis; it can thus explain the recovery of lower numbers of living cells than at start, and may also lead to an underestimation of cell expansion in conditions where higher numbers are recovered at the end of culture. Neonatal beta cells thus appear more susceptible to glucose toxicity. They were also much more susceptible to GKA-induced toxicity: in presence of GKA 3 µm, >50% dead cells were counted in the neonatal preparation at day 15, at all glucose concentrations, whereas no GKA-toxicity was observed in 8 wk-beta cells cultured at 5 mmol/l and only a minor one at higher glucose levels (data not shown).

## Discussion

Reduced beta cell mass is a hallmark of type 1 diabetes and a clinically aggravating factor in type 2 diabetes. It therefore became the target of projects searching for agents that can increase the number of beta cells in patients. Although several in vivo and in vitro conditions have been reported to induce beta cell proliferation [31], [32], their implementation to increase beta cell mass in diabetic models has been limited and variable. There are, so far, virtually no published data on compounds that increase the absolute number of primary beta cells in vitro. In order to identify compounds with such effect, we developed a method for following the number of living beta cells during culture. It is coupled to measurements of the percent beta cells in proliferative activity and the percent dead cells so that the relative contribution of both processes can be assessed. Cell number and characteristics are quantified by whole-well imaging. In the present study, the assay is conducted on purified single rat beta cells under conditions that maintain their survival for at least two weeks in absence of growth and survival factors from serum and of influences by other cell types as occur in isolated islet preparations; while this model allows to identify direct effects on beta cells their physiologic relevance will have to be subsequently addressed in animal models.

Glucose is since long considered to exert influences on the beta cell mass [11], [12]. The nutrient has been reported to positively or negatively influence the survival [3], [6], [8], [33] and/or the proliferation [1], [9], [27], [32], [34] of beta cells. Variability in its effects might be attributable to differences in concentration and duration, and in environmental conditions but also to differences in read-out parameters; data on direct effects on total beta cell numbers are however lacking. The present in vitro study shows that glucose can increase the number of beta cells that have been purified from young-adult rats. This effect involves a time- and dose-dependent activation of beta cells into DNA-synthesis, recruiting, over a two-week culture period up to 34 percent of the cells and leading to a 30 percent increase in numbers of living cells. It was not observed at basal 5 mmol/l glucose, a condition in which beta cell numbers decrease as result of inadequate protein and mitochondrial activities [8], [34]. The observed increases in living beta cell numbers at 10 and 20 mmol/l are thus the result of two effects, a glucose-induced preservation of beta cell survival and a glucose-induced proliferation. The expanded beta cell population did not exhibit obvious differences in its phenotype: mRNA expression of the beta cell characteristic proteins Ins1, Ins2, Nkx6.1, Glut2 and glucokinase was similar to the population before replication, the average cellular insulin content was not decreased, neither the insulin producing capacity as judged by the amount of insulin released over the preceding 72 hours and expressed per million beta cells. The data do not support the concept that “switch factors” regulate a shift between insulin production and proliferation [35], but do not exclude it in view of the fact that they were collected for the entire beta cell population whereas only 20 to 30 percent of the cells appeared recruited into a replicative activity.

Only a fraction of the beta cells from young-adult rats underwent replication during the two-week culture. Its recruitment was not synchronous but presented intercellular differences in time to activation. This dissimilarity in glucose-induced proliferation is another illustration of the functional heterogeneity in the beta cell population [15]. It should nevertheless be distinguished from the previously reported intercellular differences in glucose-induced insulin synthesis. While the latter can, to a large extent, be overcome by several days of culture at high glucose [15], this was not the case for activation of proliferation, with a majority of cells remaining unresponsive during the two-week study period. We examined whether responsive cells belonged to a subpopulation which, when activated into proliferation, would proceed into successive cell cycles. Using sequential EdU-BrdU labeling, this appeared not the case: beta cells entering a second cycle were rare. Glucose-induced replication thus resulted from recruitment of quiescent beta cells into the cell cycle instead of activating a limited pool into repetitive replication. In fact, subsequent re-entry into a new cycle was restricted by a “refractory” period, making a post-mitotic beta cell less likely to replicate than a resting beta cell. While our data do not support the existence of a limited replicative pool of beta cells [36], they neither demonstrate that all beta cells exhibit the same potential to proliferate [26], [27], [37]. The latter is however not excluded since it may depend on synergistic stimuli as was also noticed for glucose-induced insulin release [38].

Beta cell proliferation appears to be regulated by the rate of glycolysis [13], [27], [39]. Our in vitro study confirms this view and shows that GKA reduces the threshold for glucose-induced recruitment of young-adult beta cells into the cell cycle. However, the maximal number of glucose-recruitable beta cells did not increase, suggesting that other factors or mechanisms are responsible for the non-replicative state in a majority of the cells; this percentage appears to be higher during longer culture periods in the presence of serum [40], [41]. We have not examined whether GKA shortens the post-replication quiescence period between consecutive cell cycles [27]. Our study was also not intended to investigate the mechanism through which glucose metabolism induces proliferation in the recruited cells. It has been reported that glucose-induced modulation of ATP-sensitive potassium channels and membrane depolarization [13], [42] might be permissive for the mitogenic effects of glucose. Glucose was also shown to up-regulate insulin receptor substrate 2 expression, which could initiate downstream signaling cascades leading to activation of Akt/PKB and nuclear exclusion of FoxO1 [42], [43], [44], [45]. Additionally, a recent study has demonstrated that proliferative response of human and rodent beta cells to glucose correlates with the expression level of carbohydrate response element-binding protein, a pleiotropic glucose-sensing transcription factor [46]. Use of the 804G matrix coating has been reported to improve beta cell survival and proliferation [40], [41].

Beta cells isolated from old rats did not undergo the changes observed in young-adult beta cells. They appeared not more susceptible to cell death during culture at low glucose (5 mmol/l) as if this concentration was sufficient as survival factor. They were also not stimulated by 10 and 20 mmol/l to an increase in cell number, either because their phenotype resists recruitment into proliferative activity or became less glucose sensitive [10], [27], [36], [47], [48], [49], [50], [51], [52], [53]. On the other hand, neonatal beta cells replicated in absence of elevated glucose concentrations, and to a much higher extent than young-adult beta cells during glucose activation; their number doubled within two weeks. The majority of neonatal cells appeared primed to proliferate at basal 5 mmol/l glucose without further increase at 10 mmol/l. Proliferating neonatal beta cells did not exhibit a refractory period, which is at variance with young-adult cells; they can thus re-enter a new cell cycle as rapidly as quiescent cells. Our data are not indicative for the presence of a specialized pool of continuously replicating beta cells, but clearly indicate a major shift in the conditions under which beta cells proliferate between birth and young-adult age, i.e. from a population where most cells replicate at basal glucose to one where a minority of cells replicates at elevated glucose concentrations. A delay or acceleration in this transition might have important implications on the size of the beta cell population later in life. These observations also underline the need to further compare phenotypes and metabolic pathways in beta cells in this time window [30], [54], [55].

Neonatal beta cells have been reported to lack the glucose responsiveness as seen in adult beta cells [24], [54], [56]. They appear on the other hand much more susceptible to glucotoxicity, which explains the failure to expand their population at 20 mmol/l glucose. When cultured at this concentration, a progressive increase in the number of dead cells was noticed, leading to a final number of living beta cells that was 20 percent lower than at start. This observation might have pathophysiologic relevance as it raises the possibility that beta cells early in life are particularly susceptible to metabolic alterations, with consequences on their survival and on the size of the developing beta cell mass.

## Supporting Information

Figure S1 **Young-adult and neonatal rat beta cells differ in their post-mitotic refractory period.** Comparison of the predicted (white bars) and counted (black bars) percentages of EdU^+^BrdU^+^ beta cells from adult and neonatal rats, by assuming stochastic recruitment of beta cells into the cell cycle. Cells were cultured at indicated glucose concentration and labeled with EdU and BrdU as described in Methods. Data are expressed as means ± SEM; *, p<0.05, n = 3-5.
(TIF)

Figure S2 **Stability in beta-cell characteristic genes following glucose-induced recruitment of young adult beta cells into proliferative activity.** Beta cells purified from 8 week-old rats were cultured for 15 days at 10 mmol/l glucose. The mRNA levels of Ins1, Ins2, Pdx1, Nkx6.1, PCSK1, Glut2 and Glucokinase (Gck) were quantified by qPCR and expressed relative to the levels in freshly purified cells (means ± SEM for n = 4; no statistically significant differences detected).
(TIF)

Figure S3 **Comparison of beta-cell characteristic genes in preparations isolated from neonatal, 8-week and 40-week old rats.** mRNA expression levels of Gck, NeuroD1, MafA, Nkx6.1, Pdx1, Glut2, Ins1, Ins2 were measured by qPCR, and represented relative to the PSMC5 mRNA level of the preparation under study, i.e. beta cells from neonatal (white bars), 8-week old (black bars) and 40-week old (grey bars) rats. Columns represent means ± SEM which are statistically compared to values for 8-week old rats: **, p<0.01; ***, p<0.001.
(TIF)

Table S1 **Effect of age on susceptibility of rat beta cells to glucose toxicity.** Beta cells purified from neonatal (n = 3), 8 week (n = 8) and 40 week (n = 4) old rats were cultured for 15 days at the indicated glucose concentrations. The percent dead beta cells was determined by the propidium iodide assay and expressed as means ± SEM; statistical significance of differences were calculated by two-tailed unpaired Student's t-test: #, p<0.001 versus 10 mmol/l glucose for same age group; *, p<0.05; **, p<0.01; ***, p<0.001 versus beta cells from 8 wk-old rats cultured at same glucose concentration.
(DOC)
