# Supplementary figures and images for: Glucose Regulates Rat Beta Cell Number through Age-Dependent Effects on Beta Cell Survival and Proliferation
Source: PLoS One. 2014 Jan 9;9(1):e85174. doi: 10.1371/journal.pone.0085174 (PMC3887027; doi:10.1371/journal.pone.0085174)

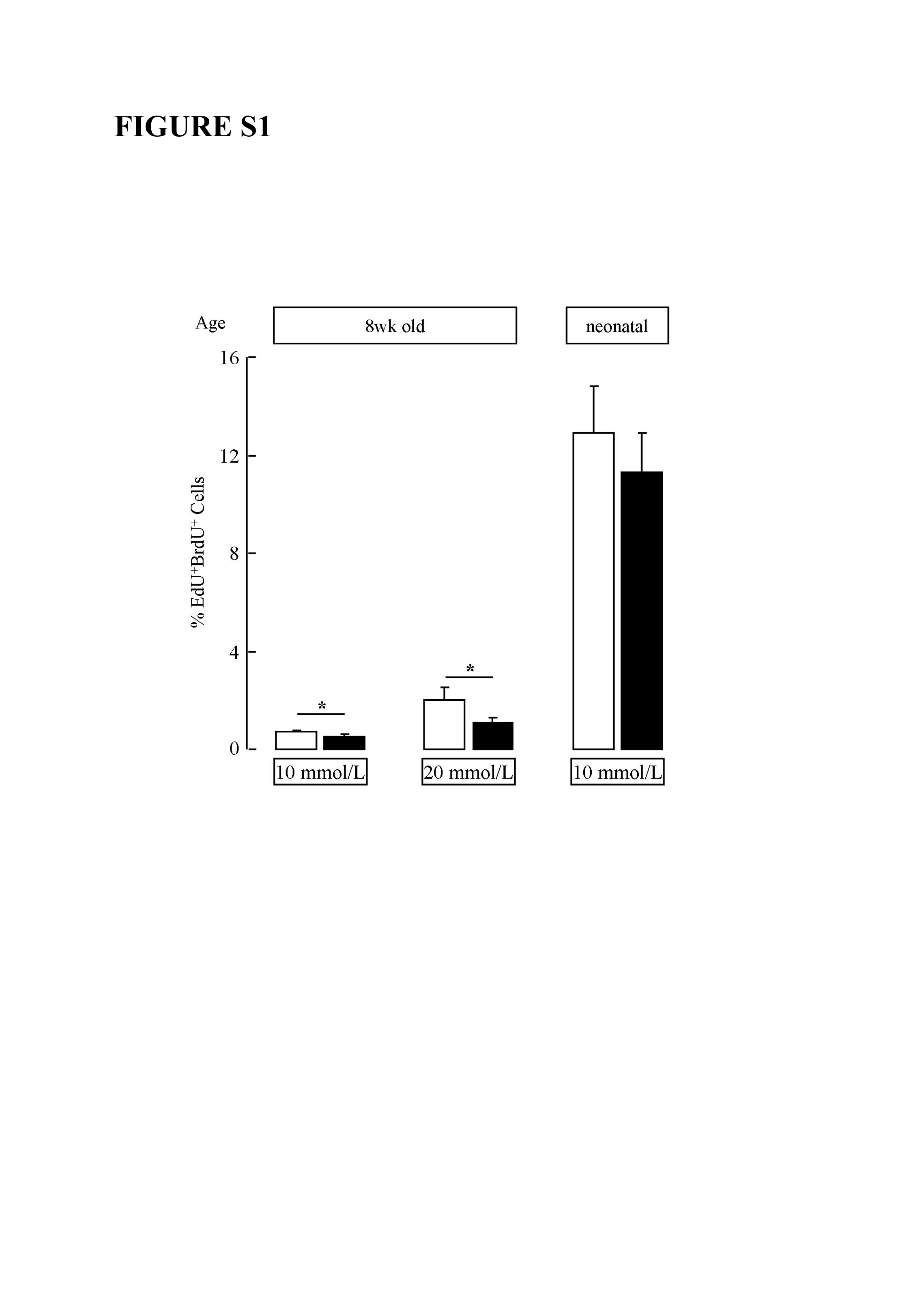

Supplement: Figure S1 — Young-adult and neonatal rat beta cells differ in their post-mitotic refractory period. Comparison of the predicted (white bars) and counted (black bars) percentages of EdU+BrdU+ beta cells from adult and neonatal rats, by assuming stochastic recruitment of beta cells into the cell cycle. Cells were cultured at indicated glucose concentration and labeled with EdU and BrdU as described in Methods. Data are expressed as means ± SEM; *, p<0.05, n = 3-5. (TIF) [file pone.0085174.s001.tif]

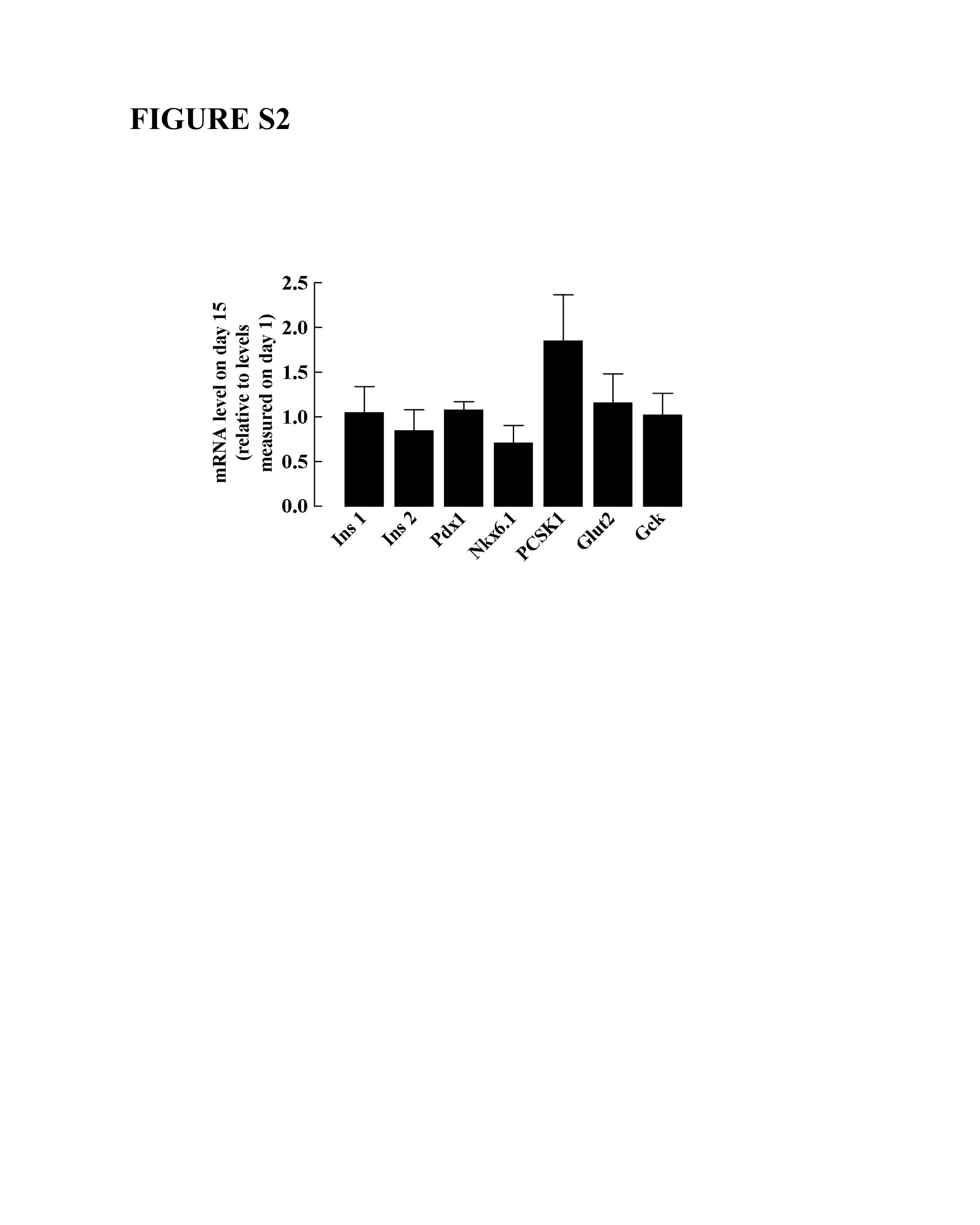

Supplement: Figure S2 — Stability in beta-cell characteristic genes following glucose-induced recruitment of young adult beta cells into proliferative activity. Beta cells purified from 8 week-old rats were cultured for 15 days at 10 mmol/l glucose. The mRNA levels of Ins1, Ins2, Pdx1, Nkx6.1, PCSK1, Glut2 and Glucokinase (Gck) were quantified by qPCR and expressed relative to the levels in freshly purified cells (means ± SEM for n = 4; no statistically significant differences detected). (TIF) [file pone.0085174.s002.tif]

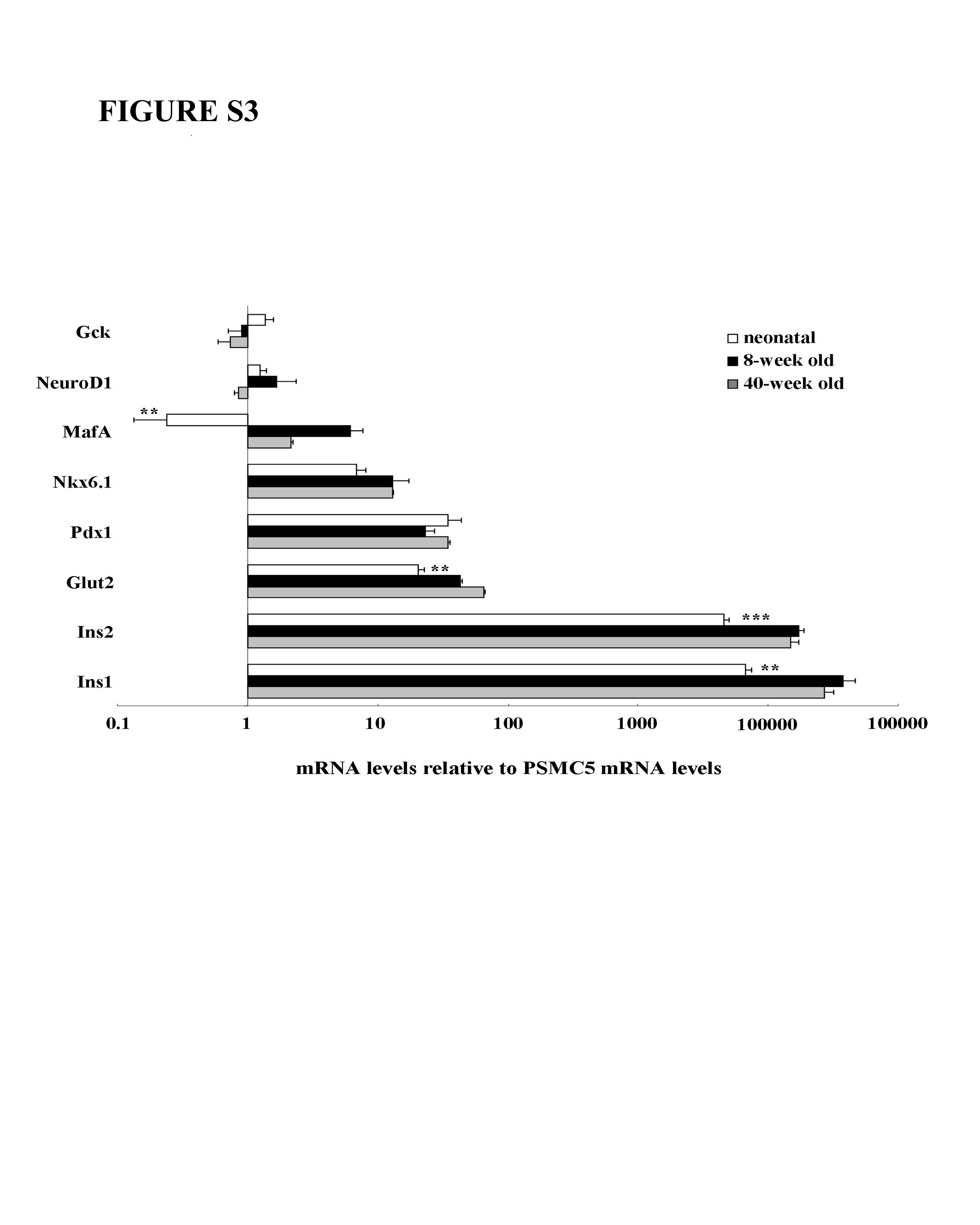

Supplement: Figure S3 — Comparison of beta-cell characteristic genes in preparations isolated from neonatal, 8-week and 40-week old rats. mRNA expression levels of Gck, NeuroD1, MafA, Nkx6.1, Pdx1, Glut2, Ins1, Ins2 were measured by qPCR, and represented relative to the PSMC5 mRNA level of the preparation under study, i.e. beta cells from neonatal (white bars), 8-week old (black bars) and 40-week old (grey bars) rats. Columns represent means ± SEM which are statistically compared to values for 8-week old rats: **, p<0.01; ***, p<0.001. (TIF) [file pone.0085174.s003.tif]
