# Supplementary material for: Glucose Regulates Rat Beta Cell Number through Age-Dependent Effects on Beta Cell Survival and Proliferation
Source: PLoS One. 2014 Jan 9;9(1):e85174. doi: 10.1371/journal.pone.0085174 (PMC3887027; doi:10.1371/journal.pone.0085174)
Supplement: Table S1 — Effect of age on susceptibility of rat beta cells to glucose toxicity. Beta cells purified from neonatal (n = 3), 8 week (n = 8) and 40 week (n = 4) old rats were cultured for 15 days at the indicated glucose concentrations. The percent dead beta cells was determined by the propidium iodide assay and expressed as means ± SEM; statistical significance of differences were calculated by two-tailed unpaired Student's t-test: #, p<0.001 versus 10 mmol/l glucose for same age group; *, p<0.05; **, p<0.01; ***, p<0.001 versus beta cells from 8 wk-old rats cultured at same glucose concentration. (DOC) [file pone.0085174.s004.doc]

**Table S1: Effect of age on susceptibility of rat beta cells to glucose toxicity.**

|  | Percent Dead Beta Cells on Day 15 | | |
| --- | --- | --- | --- |
| Age (weeks) | 0 | 8 | 40 |
| Culture 15 days |  |  |  |
| 5mmol/l glucose | 12 ± 2 | 17 ± 3# | 8 ± 1* |
| 10mmol/l glucose | 16 ± 1*** | 4 ± 1 | 8 ± 4 |
| 20mmol/l glucose | 31 ± 4*** | 8 ± 1 | 13 ± 4 |

Beta cells purified from neonatal (n=3), 8 week (n=8) and 40 week (n=4) old rats were cultured for 15 days at the indicated glucose concentrations. The percent dead beta cells was determined by the propidium iodide assay and expressed as means ± SEM; statistical significance of differences were calculated by two-tailed unpaired Student’s *t*-test: #, p<0.001 versus 10mmol/l glucose for same age group; *, p<0.05; **, p<0.01; ***, p<0.001 versus beta cells from 8 wk-old rats cultured at same glucose concentration.
